# Supplementary figures and images for: Biochemical, biomarker, and behavioral characterization of the GrnR493X mouse model of frontotemporal dementia
Source: bioRxiv. 2023 May 29:2023.05.27.542495. Preprint. [Version 1] doi: 10.1101/2023.05.27.542495 (PMC10312473; doi:10.1101/2023.05.27.542495)

6 months

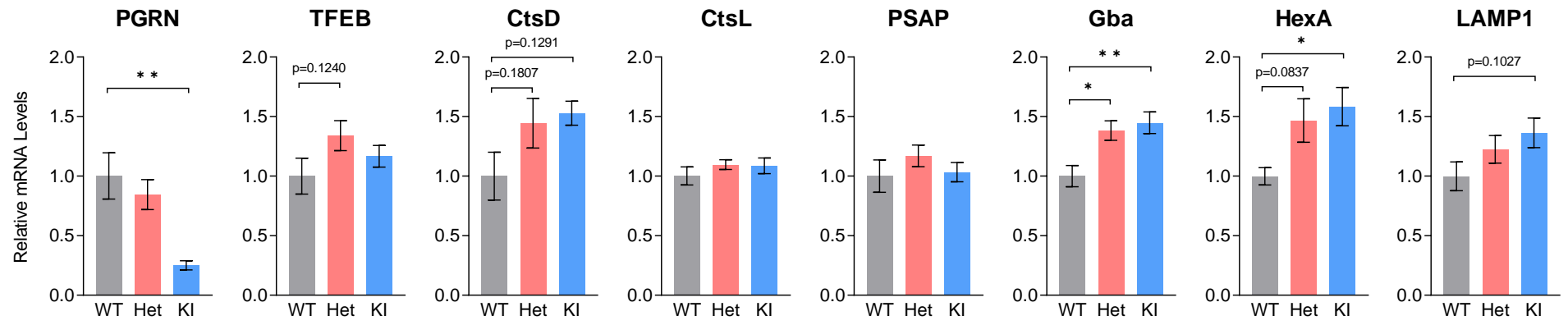

12 months

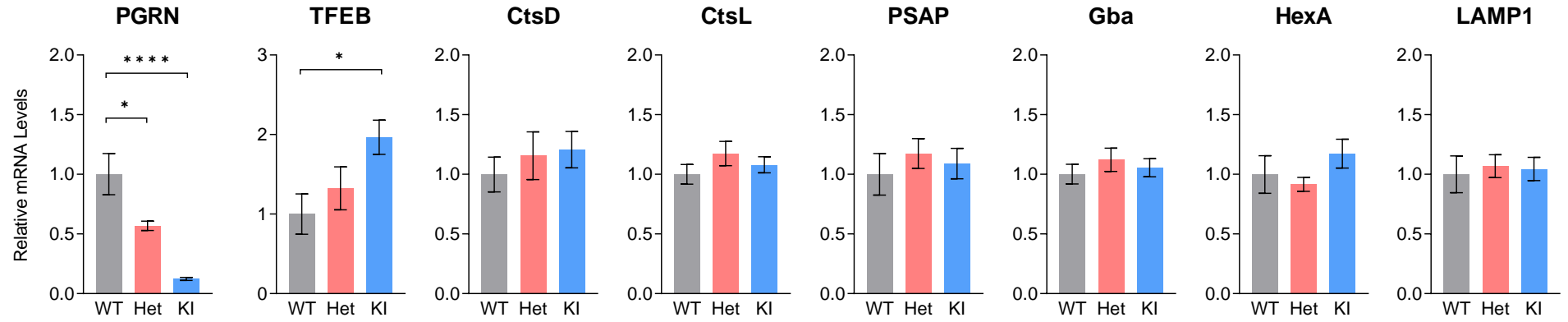

Figure S1

Supplement: Supplement 1 — Figure S1. Grn mice have increased expression of several lysosomal genes in the thalamus. Quantification of mRNA levels by RT-qPCR. Bars represent mean ± SEM (n = 5–8 per group, male and female mice); * p < 0.05, ** p < 0.01, and **** p < 0.0001 compared to wildtype group, as determined by one-way ANOVA with Dunnett post hoc test. WT, wild-type; Het, Grn+/R493X heterozygous mice; KI, GrnR493X/R493X knockin mice. [file media-1.pdf]

**A**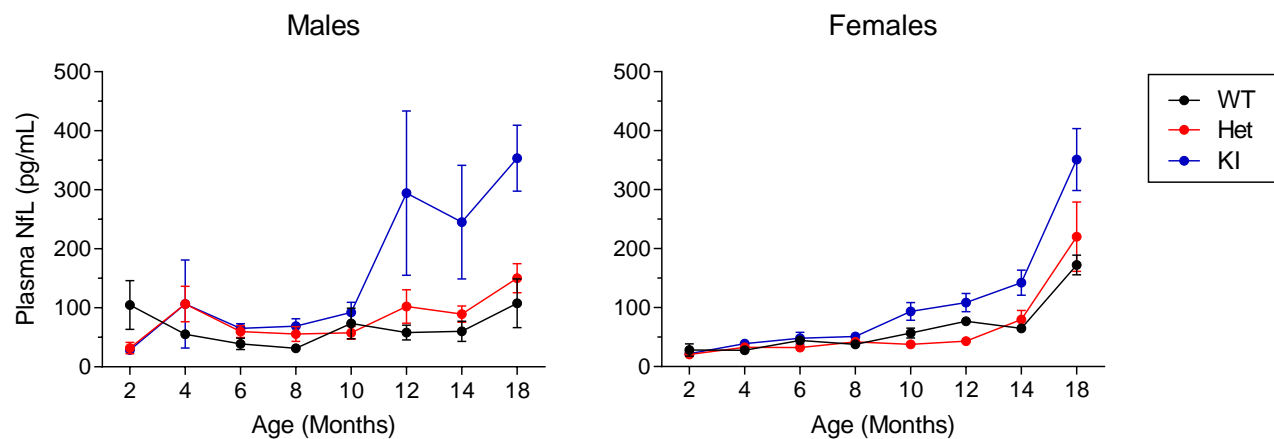**B**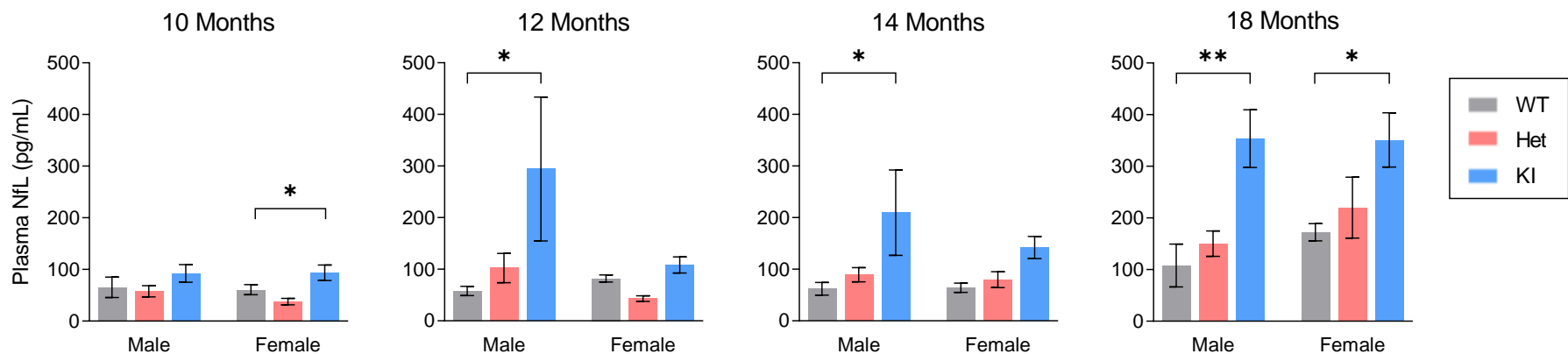**C**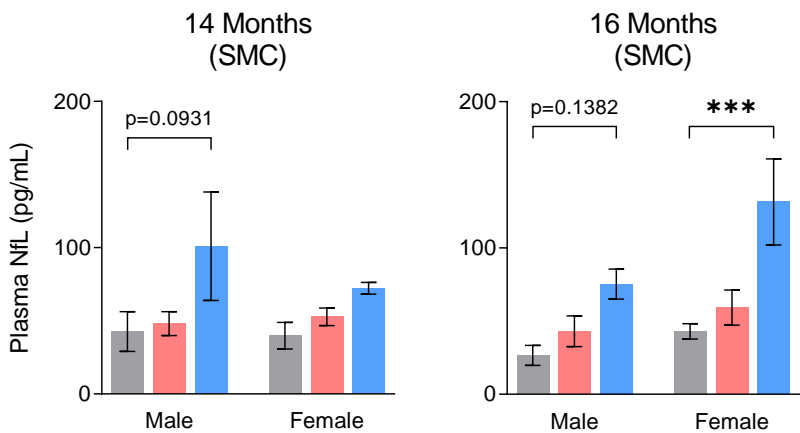**D**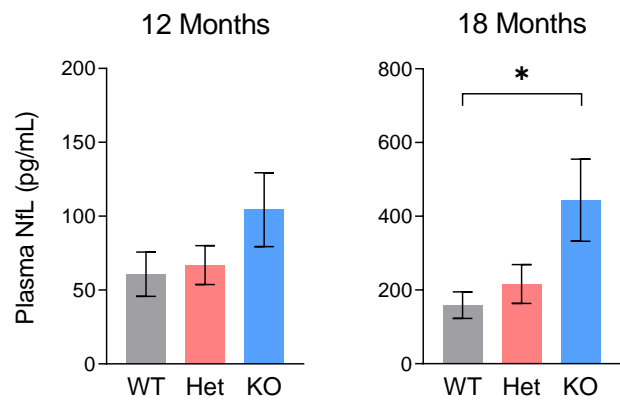**Figure S2**

Supplement: Supplement 2 — Figure S2. Homozygous GrnR493X mice, but not heterozygous mice, have increased plasma NfL levels. A-B) Plasma NfL levels at different ages and separated by sex measured using the Quanterix Simoa platform (n = 3–7 per group for male mice, n = 4–5 per group for female mice). C) Plasma NfL levels at different ages and separated by sex measured using the Sigma SMC platform (n = 3–6 per group for male mice, n = 3–4 per group for female mice). D) Plasma NfL levels in Grn knockout mouse model measured using the Quanterix Simoa platform (n = 4–6 per group at 12 months old, n = 6–8 per group at 18 months old, male and female mice). Bars represent mean ± SEM; * p < 0.05 and ** p < 0.01 compared to wild-type group, as determined by one-way ANOVA with Dunnett post hoc test. WT, wild-type; Het, Grn+/R493X heterozygous mice; KI, GrnR493X/R493X knockin mice. [file media-2.pdf]

**A**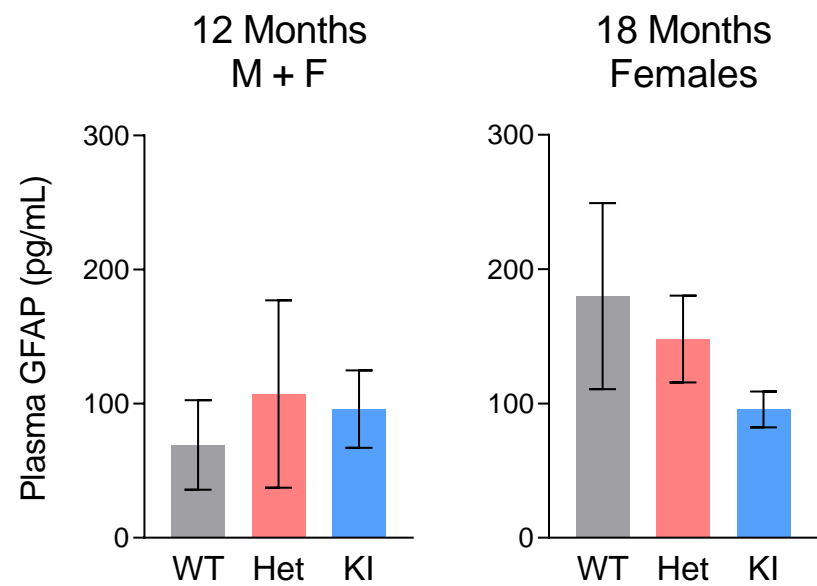**B**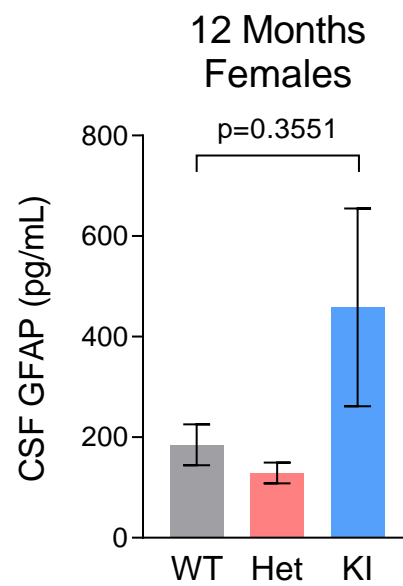**Figure S3**

Supplement: Supplement 3 — Figure S3. Plasma and CSF levels of GFAP in GrnR493X mice. A) Plasma GFAP levels (n = 67 per group at 12 months old, male and female mice; n = 4 per group at 18 months old, female mice only). B) CSF GFAP levels (n = 3–4 per group, female mice only). Bars represent mean ± SEM; statistical analysis was performed by one-way ANOVA with Dunnett post hoc test. WT, wild-type; Het, Grn+/R493X heterozygous mice; KI, GrnR493X/R493X knockin mice. [file media-3.pdf]

**A****Open Field Activity**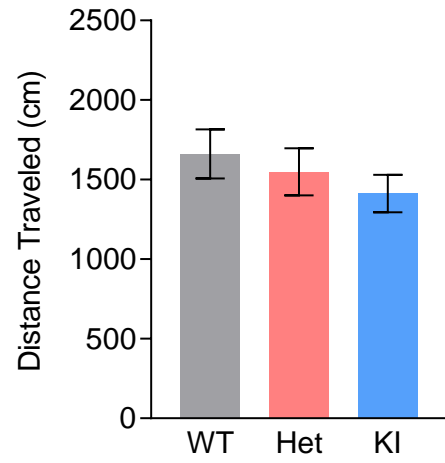**B****Center of Open Field**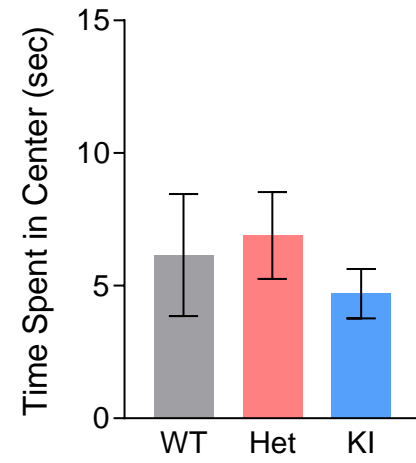**C****Forced Swim Test**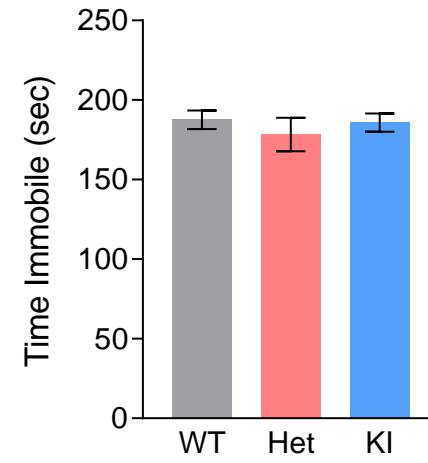**D****Nest Building**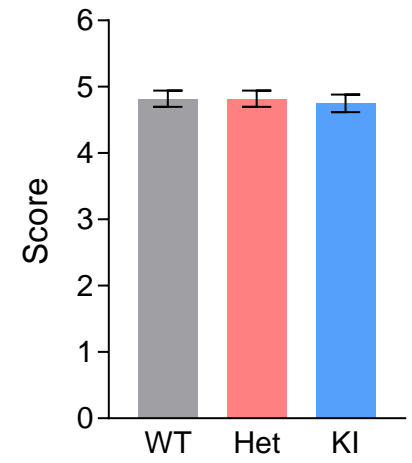**Figure S4**

Supplement: Supplement 4 — Figure S4. GrnR493X mice show no differences in other behavioral tests performed. Bars represent mean ± SEM (n= 8–12 per group, male mice only, 11 months old); statistical analysis was performed by one-way ANOVA with Dunnett post hoc test. WT, wild-type; Het, Grn+/R493X heterozygous mice; KI, GrnR493X/R493X knockin mice. [file media-4.pdf]
