# Supplementary material for: Biochemical, biomarker, and behavioral characterization of the GrnR493X mouse model of frontotemporal dementia
Source: bioRxiv. 2023 May 29:2023.05.27.542495. Preprint. [Version 1] doi: 10.1101/2023.05.27.542495 (PMC10312473; doi:10.1101/2023.05.27.542495)
Supplement: Supplement 5 [file media-5.docx]

**Table S1. qPCR primer sequences.**

| **Gene** | **Forward Primer** | **Reverse Primer** |
| --- | --- | --- |
| 36B4 | CACTGGTCTAGGACCCGAGAAG | GGTGCCTCTGAAGATTTTCG |
| Cyclo | GGCTCCGTCGTCTTCCTTTT | ACTCGTCCTACAGATTCATCTCC |
| PGRN | TGGTTCACACACGATGCGTTTCAC | AAAGGCAAAGACACTGCCCTGTTG |
| Tfeb | CCACCCCAGCCATCAACAC | CAGACAGATACTCCCGAACCTT |
| CtsD | CCTGGCTTCGTCCTCCTTC | GGCGATGACTGCATGGAGT |
| CtsL | ATCAAACCTTTAGTGCAGAGTGG | CTGTATTCCCCGTTGTGTAGC |
| PSAP | CCTGTCCAAGACCCGAAGAC | CAAGGAAGGGATTTCGCTGTG |
| Gba | GACCAACGCTTGCTGCTAC | ACAGCAATGCCATGAACGTA |
| Hexa | ACCTGGGAGGGGATGAAGT | ATGAAGGCCTGGATGTTGG |
| LAMP1 | GCCCACAAACCCCACTGTAT | TTTGGGCTGATGTTGAACGC |
| Iba1 | ATCAACAAGCAATTCCTCGATGA | CAGCATTCGCTTCAAGGACATA |
| GFAP | CGGAGACGCATCACCTCTG | AGGGAGTGGAGGAGTCATTCG |
| TNFα | ACGGCATGGATCTCAAAGAC | AGATAGCAAATCGGCTGACG |
| IL-1β | GCTTCAGGCAGGCAGTATC | AGGATGGGCTCTTCTTCAAAG |
| MCP1 | CTTCCTCCACCACCATGCA | CCAGCCGGCAACTGTGA |
| C1qa | AAAGGCAATCCAGGCAATATCA | TGGTTCTGGTATGGACTCTCC |
| C3 | CCAGCTCCCCATTAGCTCTG | GCACTTGCCTCTTTAGGAAGTC |
